# Proteomics reveals the effects of drought stress on the kernel development and starch formation of waxy maize

**DOI:** 10.1186/s12870-021-03214-z

**Published:** 2021-09-23

**Authors:** Jian Guo, Lingling Qu, Yifan Hu, Weiping Lu, Dalei Lu

**Affiliations:** 1 Jiangsu Key Laboratory of Crop Genetics and Physiology, Jiangsu Key Laboratory of Crop Cultivation and Physiology, Agricultural College of Yangzhou University, Yangzhou, 225009 P. R. China; 2 Jiangsu Co-Innovation Center for Modern Production Technology of Grain Crops, Yangzhou University, Yangzhou, 225009 P. R. China; 3 Joint International Research Laboratory of Agriculture and Agri-Product Safety of the Ministry of Education, Yangzhou University, Yangzhou, 225009 P. R. China

**Keywords:** Waxy maize, Drought stress, Kernel development, Proteome

## Abstract

**Background:**

Kernel development and starch formation are the primary determinants of maize yield and quality, which are considerably influenced by drought stress. To clarify the response of maize kernel to drought stress, we established well-watered (WW) and water-stressed (WS) conditions at 1–30 days after pollination (dap) on waxy maize (*Zea mays* L. *sinensis Kulesh*).

**Results:**

Kernel development, starch accumulation, and activities of starch biosynthetic enzymes were significantly reduced by drought stress. The morphology of starch granules changed, whereas the grain filling rate was accelerated. A comparative proteomics approach was applied to analyze the proteome change in kernels under two treatments at 10 dap and 25 dap. Under the WS conditions, 487 and 465 differentially accumulated proteins (DAPs) were identified at 10 dap and 25 dap, respectively. Drought induced the downregulation of proteins involved in the oxidation–reduction process and oxidoreductase, peroxidase, catalase, glutamine synthetase, abscisic acid stress ripening 1, and lipoxygenase, which might be an important reason for the effect of drought stress on kernel development. Notably, several proteins involved in waxy maize endosperm and starch biosynthesis were upregulated at early-kernel stage under WS conditions, which might have accelerated endosperm development and starch synthesis. Additionally, 17 and 11 common DAPs were sustained in the upregulated and downregulated DAP groups, respectively, at 10 dap and 25 dap. Among these 28 proteins, four maize homologs (i.e., A0A1D6H543, B4FTP0, B6SLJ0, and A0A1D6H5J5) were considered as candidate proteins that affected kernel development and drought stress response by comparing with the rice genome.

**Conclusions:**

The proteomic changes caused by drought were highly correlated with kernel development and starch accumulation, which were closely related to the final yield and quality of waxy maize. Our results provided a foundation for the enhanced understanding of kernel development and starch formation in response to drought stress in waxy maize.

**Supplementary Information:**

The online version contains supplementary material available at 10.1186/s12870-021-03214-z.

## Background

Drought is the most serious environmental stress that obstructs crop growth and production worldwide and become more frequent due to climate change [1]. Maize (*Zea mays* L.) is one of the most important cereal crops, and maize production is frequently compromised because of the increasing frequency and intensity of drought [2]. Drought stress (DS) has drastically reduced maize production in many regions around the world, such as China and the United States [2, 3]. DS leads to abnormal embryo development and decrease kernel numbers, grain yield and quality, especially during the critical period of maize kernel formation [4, 5]. Thus, investigating the mechanism of drought response at the maize grain-filling stage is helpful for improving maize yield and quality under DS.

The development of endosperm (i.e., early development, differentiation, and maturation) has a remarkable influence on grain weight and quality and is highly sensitive to DS [6]. DS reduces cell viability and accelerates nuclear deformation and programmed cell death in wheat endosperm cells [7]. DS occurs during early endosperm development and inhibits kernel growth by decreasing endosperm cell division [8]. In the endosperm of crops, the primary function of endosperm cells is to synthesize starch and store proteins [9]. Maize endosperm, which is the foremost kernel component that determines grain weight and quality, contains about 75% starch. However, the biosynthesis and accumulation of starch are seriously threatened by DS [10]. For instance, DS has a negative effect on the development of endosperm starch granules and the composition and physicochemical properties of starches [11]. Studies reported that DS during the grain-formation stage reduced the number of endosperm cells and starch granules, but increased the starch granule size [5, 12]. Water shortages accelerate grain-filling and ripening periods and reduce the total starch accumulation, which is directly correlated with grain productivity [13, 14]. During grain development, DS impedes starch synthesis by reducing the enzymatic activities related to starch biosynthesis, which ultimately leading to a decline in grain yield [15, 16]. These enzymes mainly include: (1) Sucrose phosphate synthase (SPS) is a key enzyme in the regulation of sucrose biosynthesis in source tissues [17]. (2) Sucrose synthase (SUS) catalyzes the cleavage of sucrose, which is the first step in the conversion of sucrose to starch. (3) Adenosine diphosphate-glucose pyrophosphorylase (AGPase) catalyzes ADP-Glucose, which is the rate-limiting enzyme in starch biosynthesis. (4) Soluble starch synthase (SSS) catalyzes the glucosyl moiety of ADP-Glucose to synthesize amylose and amylopectin. (5) Starch branching enzyme (SBE) catalyzes α-(1,6)-linkages within the polymer to form the branched structure of amylopectin [18]. The study of how endosperm and starch development respond to DS is crucial to ensure the high yield and quality of maize under adverse conditions. However, our understanding of drought response mechanisms during the grain-filling stage remains unclear.

Recent advances in high-throughput technologies enable the quantitation of abundance changes in transcriptomics, proteomics, and other omics under DS [19]. Generally, many studies on the response of maize to DS focus on large-scale transcriptomic analyses [20–23]. However, transcriptome profiling has limitations because the changes in gene expression levels do not correspond directly to protein expression levels due to post-transcriptional and post-translational modifications [24]. Proteomics, as a research of post-genome era, is a crucial link between transcriptomics and metabolomics [25]. High-throughput proteomics has become a powerful tool that can provide new insights into how plants respond to DS at the protein level [26]. Proteins are important for plant DS response because of their direct effect on metabolism and other cellular processes [27]. For example, alterations in proteins related to the metabolism of carbohydrates and antioxidation and defense systems are found to be characteristic features between drought-tolerant and drought-sensitive wheat [28]. In maize, the changes in seeding leaf protein abundance are consistent with the observed phenotypes of YE8112 (drought-tolerant line) and Mo17 (drought-sensitive line) under DS [29]. Moreover, comparative proteomics analysis is an effective strategy to identify pivotal candidate functional proteins and regulatory networks under DS [30]. In maize, studies on the proteome response to DS on kernel development and starch formation are limited.

Thus, in the present study, we performed the comparative proteomic analysis of waxy maize hybrid kernels under DS during the grain filling and analyzed the dynamic changes of kernel development, starch accumulation, and related enzymatic activities. The objectives of the present study were to identify candidate proteins that might play important roles in the kernel development, starch accumulation, and drought stress response. The results may enhance our understanding of the regulatory networks of endosperm development and starch formation in response to DS. Furthermore, drought responsive proteins identified herein could be harnessed for improving drought tolerance breeding in waxy maize yield and quality.

## Results

### Dynamic changes in kernel development under drought stress

**Fig. 1:**
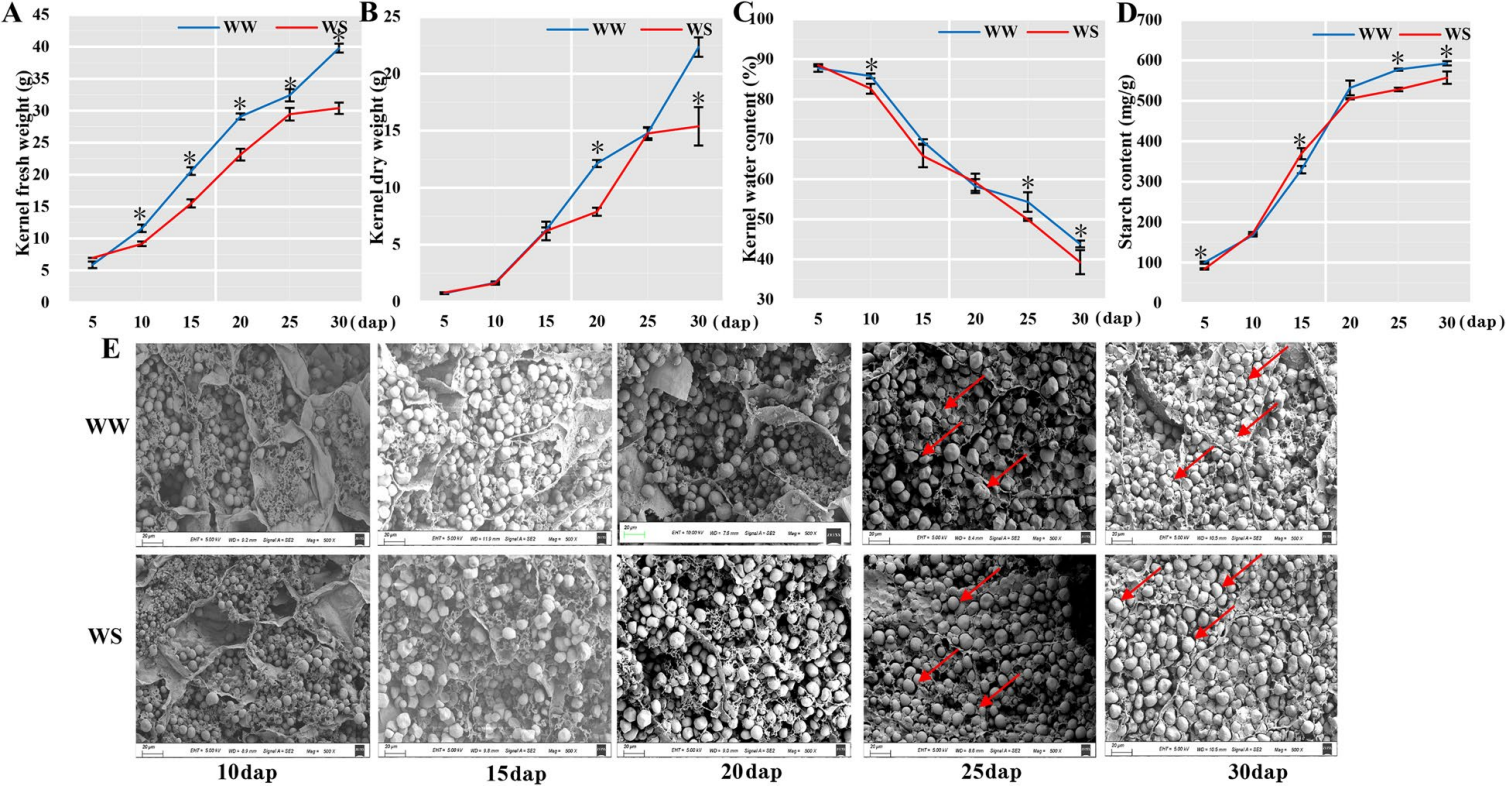
Dynamic changes on grain-filling properties, starch content, and morphological features of waxy maize kernel under WW and WS treatments. **A** Kernel fresh weight from 5 to 30 dap. **B** Kernel dry weight from 5 to 30 dap. **C** Kernel water content from 5 to 30 dap. **D** Starch content of kernels from 5 to 30 dap. **E** Scanning electron micrographs of endosperm cells under WW and WS treatments. Example of starch granules are marked with red arrows. WW: well-watered; WS: water-stressed. Asterisk denote statistical difference at *P* < 0.01 using ANOVA followed by LSD test

Ears were harvested and analyzed at six stages after pollination (from 5 to 30 dap with five days apart). Kernel fresh and dry weights gradually increased with kernel development under well-watered (WW) and water-stressed (WS) conditions. However, kernel development was significantly inhibited under WS conditions (*P* < 0.01, Fig. 1A and B). From 25 to 30 dap, the increase rates of kernel fresh and dry weights under WW conditions were significantly higher than those under WS conditions. The kernel water content gradually decreased with kernel development, and the decline rate of WS was higher than that of WW during the whole grain-filling stage (Fig. 1C). These results indicated that WS conditions accelerated grain maturity, shortened grain filling, and reduced grain weight.

### Dynamic changes in the starch accumulation and enzymatic activities under drought stress

**Fig. 2:**
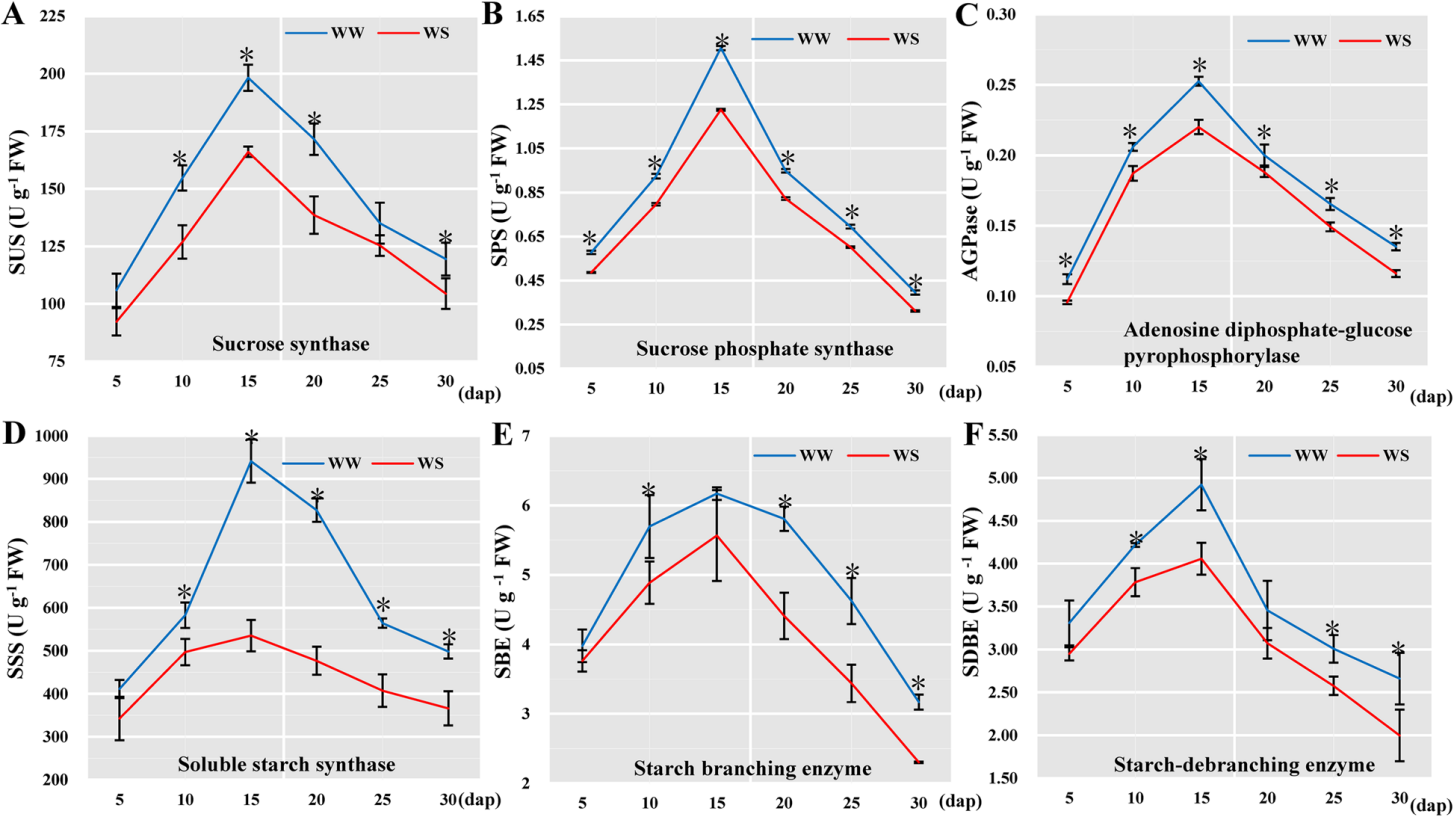
Dynamic changes in the enzymatic activities related to starch biosynthesis in maize endosperm at 5–30 dap in response to drought stress. **A** SUS: sucrose synthase. **B** SPS: Sucrose phosphate synthase. **C** AGPase: ADP-glucose pyrophosphorylase. **D** SSS: soluble starch synthase. **E** SBE: starch-branching enzyme. **F** SDBE: starch-debranching enzyme. WW: well-watered; WS: water-stressed. Asterisk denote statistical difference at *P* < 0.01 using ANOVA followed by LSD test

With the progress of grain filling, the accumulation of starch in endosperm cells increased gradually. The starch content increased sharply from 10 to 20 dap under both water levels. Our results showed no difference in the starch content under two treatments at 10 dap, but the starch content under WS treatment was significantly higher than that under WW treatment at 15 dap (Fig. 1D). Compared with WW, WS caused a significant decrease in starch accumulation from 20 to 30 dap (Fig. 1D). The starch in endosperm cells at 10–30 dap was observed and revealed by scanning electron microscopy to evaluate the morphological features and accumulation of starch granules under WW and WS conditions (Fig. 1E). The starch granule was irregularly shaped due to crowding under WW treatment at 25 and 30 dap but mostly spherical under WS (Fig. 1E). In addition, the activities of starch synthetic enzymes (i.e., AGPase, SUS, SBE, SDBE, SPS, and SSS) increased initially and declined thereafter with kernel development (Fig. 2). Furthermore, all enzymatic activities were significantly reduced under WS treatment during grain filling (*P* < 0.01). Therefore, DS significantly inhibited the kernel development, starch synthetic activities, and starch accumulation during grain filling.

### Quantitative proteomic analysis

**Fig. 3:**
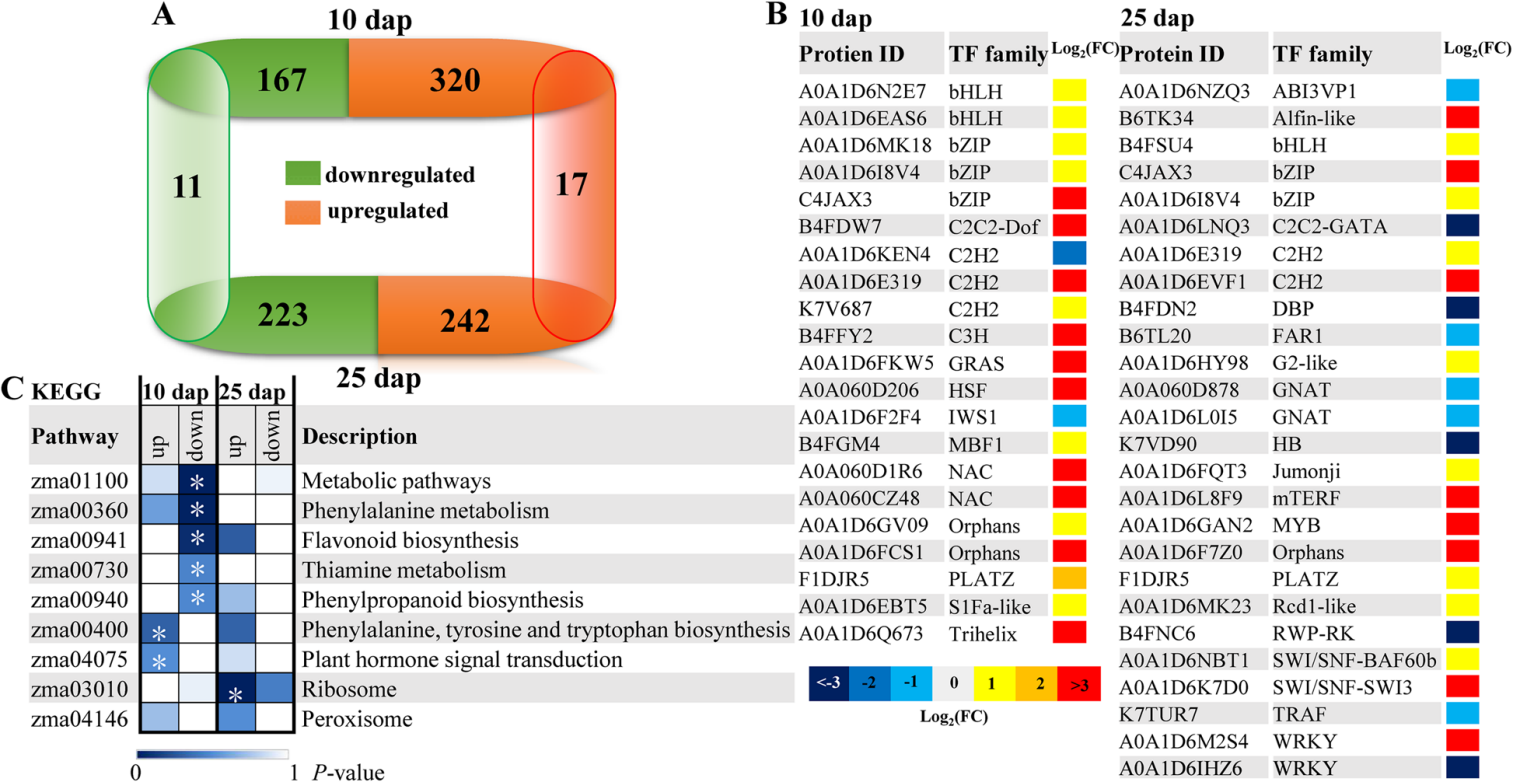
Distribution of differentially accumulated proteins (DAPs) following drought stress effects during waxy maize grain filling. **A** The number of DAPs at 10 dap and 25 dap are given. **B** Transcription factors (TFs) enriched in up- and down-regulated DAPs. The different color represented different level of log_2_(fold change). **C** KEGG pathway enrichment analysis of the up- and down-regulated DAPs [31]. The color scale at the bottom represents the significance (corrected *P*-value). Asterisks designate statistical (using Student’s *t*-test) significance *P* < 0.01

A total of 6,769 proteins were identified from waxy maize kernels by using DIA mass spectroscopy (Table S1). The principal component analysis (PCA) was performed to characterize all proteins in the three replicates at 10 dap and 25 dap under the WW and WS treatments. In the PCA plot, a clear separation was observed between WW and WS treatments at 10 dap and 25 dap, suggesting that the proteome was distinct between the two treatments (Figure S1). To identify the differentially accumulated proteins (DAPs) among treatments, two fold change and *P* < 0.05 were chosen as the criteria. Compared with the WW treatment, the numbers of DAPs for WS treatment were 487 (320 upregulated and 167 downregulated) at 10 dap, and 465 (242 upregulated and 223 downregulated) at 25 dap (Fig. 3A, Table S2). At 10 dap, 19 and 2 transcription factors (TFs) were identified in upregulated and downregulated proteins, respectively. Moreover, 10 and 15 TFs were found in upregulated and downregulated proteins, respectively, at 25 dap (Fig. 3B). These TFs belonged to 31 families, and members of the bHLH, bZIP, and C2H2 TF families were highly represented among the DAPs (Fig. 3B). In addition, we randomly chose ten genes for expression validation by using qRT-PCR. The results showed that seven genes expression level displayed the same trend with the abundance of the corresponding protein species, three genes showed opposite trends to the abundance of their corresponding proteins (Figure S2). Thus, changes in mRNA expression might not always adequately reflect corresponding protein levels.

Among all DAPs, 54 (38 upregulated and 16 downregulated) and 45 (24 upregulated and 21 downregulated) DAPs with experimentally confirmed functions [32, 33] were identified at 10 dap and 25 dap, respectively (Figure S3). These DAPs that responded to DS had different functions. For instance, A0A1D6JMR5 and A0A1D6Q9W2, which related to catalase and glutamine synthetase, respectively, were downregulated at 10 dap. Abscisic acid stress ripening 1 (B4FKG5) and lipoxygenase (A0A1D6DVH5) were downregulated at 25 dap. In addition, several DAPs, which involved in endosperm development (i.e., A0A1D6H9T3, A0A1D6GRB4, B4F832, and A0A1D6PZG8) and starch accumulation (i.e., A0A1D6EBS7, A0A1D6NUU8, A0A1D6PXQ9, and A0A1D6LVT4), were upregulated at 10 dap.

### KEGG pathway and GO annotation analyses of DAPs

KEGG pathways were classified into hierarchical categories according to the website (http://www.kegg.jp/kegg/kegg1.html). In present study, KEGG pathway analysis showed that DAPs at 10 dap were significantly enriched in the 11 KEGG pathway (such as “Plant hormone signal transduction”, “Starch and sucrose metabolism”, “Biosynthesis of secondary metabolites”, etc.), but only one significantly enriched pathway, i.e., “Ribosome” at 25 dap (*P* < 0.01; Figure S4). In addition, a high proportion of the upregulated DAPs at 10 dap were enriched in “Phenylalanine, tyrosine, and tryptophan biosynthesis” and “Plant hormone signal transduction”. A large number of downregulated DAPs at 10 dap were enriched in the “Metabolic pathways”, “Phenylalanine metabolism”, “Flavonoid biosynthesis”, “Thiamine metabolism”, and “Phenylpropanoid biosynthesis”. However, only one pathway, i.e., “Ribosome”, was enriched among the upregulated proteins at 25 dap (Fig. 3C).

**Fig. 4:**
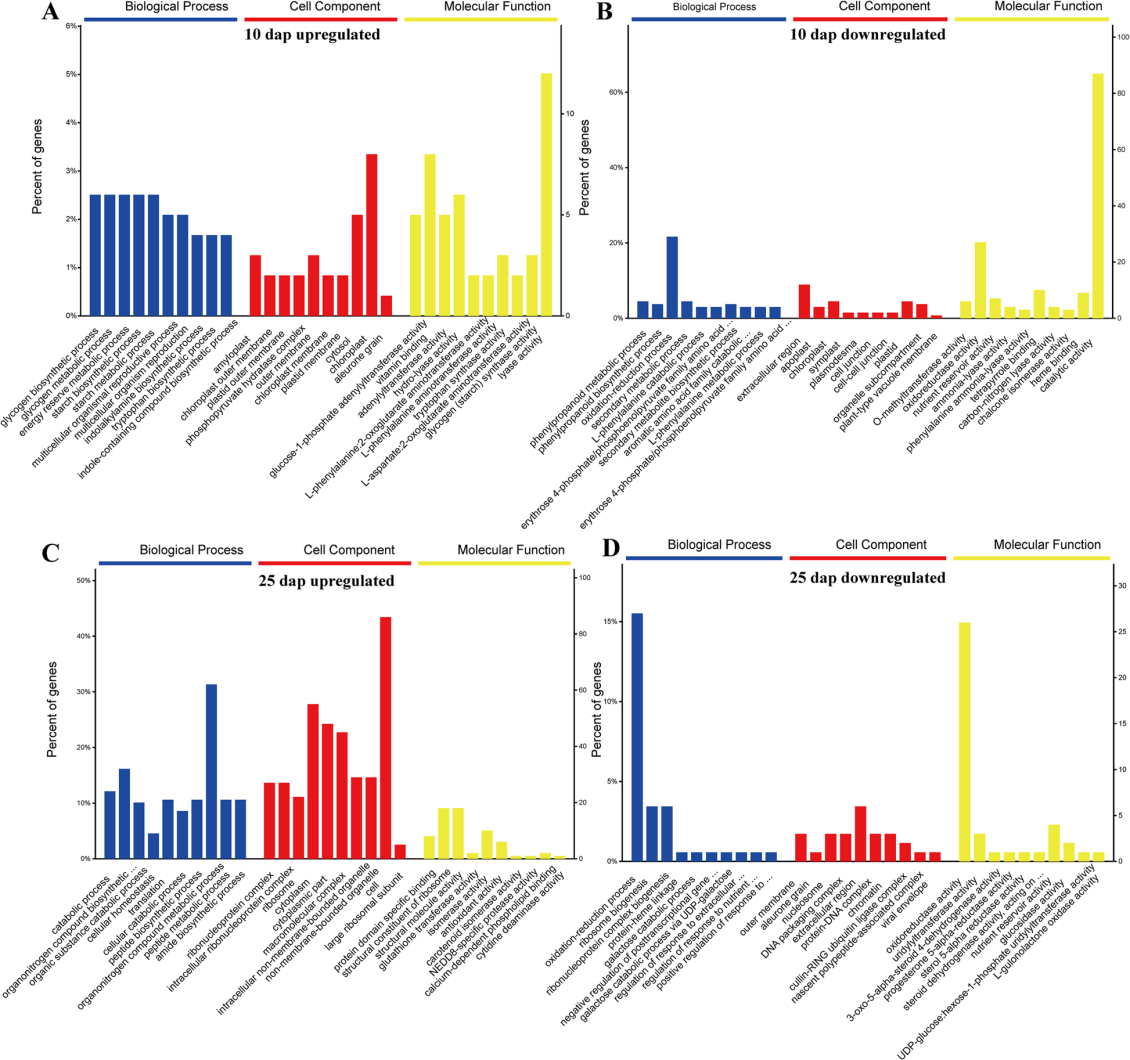
GO terms for the up- and down-regulated DAPs. **A** Upregulated DAPs at 10 dap. **B** Downregulated DAPs at 10 dap. **C** Upregulated DAPs at 25 dap. **D** Downregulated DAPs at 25 dap

We also analyzed the GO terms represented by all DAPs at two stages. The enriched GO terms at 10 dap included mainly “cellular polysaccharide metabolic process”, “polysaccharide metabolic process”, and “cellular carbohydrate metabolic process”, etc.; and at 25 dap included mainly “organonitrogen compound metabolic process”, “cellular protein metabolic process”, and “cellular nitrogen compound metabolic process”, etc. (Figure S5). These results demonstrated that the DAPs at 10 dap and 25 dap were involved in different biological processes. Additionally, in order to clarify the biological processes involved in up- and down-regulated DAPs, we performed GO analysis for these DAPs. For the upregulated DAPs at 10 dap, three significantly enriched GO terms, including “chloroplast”, “vitamin binding”, and “lyase activity”, had the most DAPs (Fig. 4A). In addition, the “oxidation–reduction process”, “oxidoreductase activity”, and “catalytic activity” had the most DAPs in downregulated DAPs at 10 dap (Fig. 4B). In the GO enrichment analysis of upregulated proteins at 25 dap, significant GO terms, i.e., the “organonitrogen compound metabolic process”, “cytoplasm”, and “cell”, contained more DAPs (Fig. 4C). Significant GO terms, i.e., “oxidation–reduction process” and “oxidoreductase activity”, had most DAPs in downregulated DAPs at 25 dap (Fig. 4D). This result suggested that the oxidation–reduction process and oxidoreductase activity were enriched in downregulated DAPs at the two stages and might negatively affect the drought tolerance of kernels under DS.

### DAPs between 10 and 25 dap

Comparison analysis was conducted for the upregulated and downregulated DAPs to discover common DAPs in response to DS among different stages. Seventeen common DAPs were sustained in the upregulated group, and 11 common DAPs were consistent in the downregulated group (Table 1). We found that the peroxidase (B4FBC8) was downregulated in the two stages. Unfortunately, these DAPs have not been reported in maize. Thus, we performed the homology comparison of these proteins through the protein–protein BLAST method and found four homologous genes with known functions in rice, including A0A1D6H543 (homolog of *PDIL1-1*), B4FTP0 (homolog of *OsADF2*), B6SLJ0 (homolog of *MODD*), and A0A1D6H5J5 (homolog of *BS1*). These genes have been shown to involved in cell wall (*BS1*), endosperm development (*PDIL1-1*), and DS response (*OsADF2* and *MODD*, Table 1) in rice. Thus, these homologous genes may have similar functions in maize.

**Table 1:** Common DAPs between 10 and 25 dap

DAPs	Protein ID	Description	Gene ID	Homologous gene in rice	Rice gene ID	Reference
Upregulated	A0A1D6G9W0	Bifunctional inhibitor/lipid-transfer protein/seed storage 2S albumin superfamily protein	Zm00001d012572			
	A0A1D6H543	Protein disulfide isomerase-like 1–2	Zm00001d016017	*PDIL1-1*	Os11g0199200	[34]
	A0A1D6HG34	Uncharacterized protein	Zm00001d017640			
	A0A1D6ICL3	Adenosine 5'-phosphosulfate reductase-like1	Zm00001d021596			
	A0A1D6KJI9	Calcium-transporting ATPase	Zm00001d031543			
	A0A1D6L6V6	Cullin-4	Zm00001d034361			
	A0A1D6L9Y9	NAD-dependent epimerase/dehydratase	Zm00001d034665			
	A0A1D6LZA4	Serine/threonine-protein phosphatase	Zm00001d037601			
	A0A1D6MHM9	RNA methyltransferase family protein	Zm00001d039477			
	B4FHI8	Hypoxia-responsive family protein	Zm00001d007153			
	B4FPE0	Cytidine/deoxycytidylate deaminase family protein	Zm00001d034726			
	B4FTP0	Actin-depolymerizing factor 6	Zm00001d013141	*OsADF2*	Os03g0780400	[35]
	B6SLJ0	Ninja-family protein 3		*MODD*	Os03g0214200	[36]
	B8A0U6	O-fucosyltransferase family protein	Zm00001d006931			
	B8A2N0	COV1	Zm00001d053350			
	K7TSG6	Rhodanese-like domain-containing protein 19 mitochondrial	Zm00001d025081			
	K7V945	EMB1374 isoform 1	Zm00001d008281			
Downregulated	A0A1D6H5J5	GDSL esterase/lipase	Zm00001d016130	*BS1*	Os02g0250400	[37]
	A0A1D6KN97	Germin-like protein	Zm00001d032047			
	A0A1D6KR63	Monocopper oxidase-like protein SKU5	Zm00001d032504			
	A0A1D6KRC1	DEAD-box ATP-dependent RNA helicase 8	Zm00001d032526			
	A0A1D6KT77	Exportin-4	Zm00001d032704			
	A0A1D6L9C7	Protein trichome birefringence-like 32	Zm00001d034621			
	B4FBC8	Peroxidase	Zm00001d052335			
	B4FEC9	Protein ESKIMO 1	Zm00001d023217			
	B6UBM9	Uncharacterized protein	Zm00001d029349			
	C0P4Y2	Pathogenesis-related family protein	Zm00001d040244			
	K7U837	F-box protein At-B	Zm00001d052850			

### Effects of DS on the accumulation level of proteins involved in maize endosperm and starch biosynthesis

**Fig. 5:**
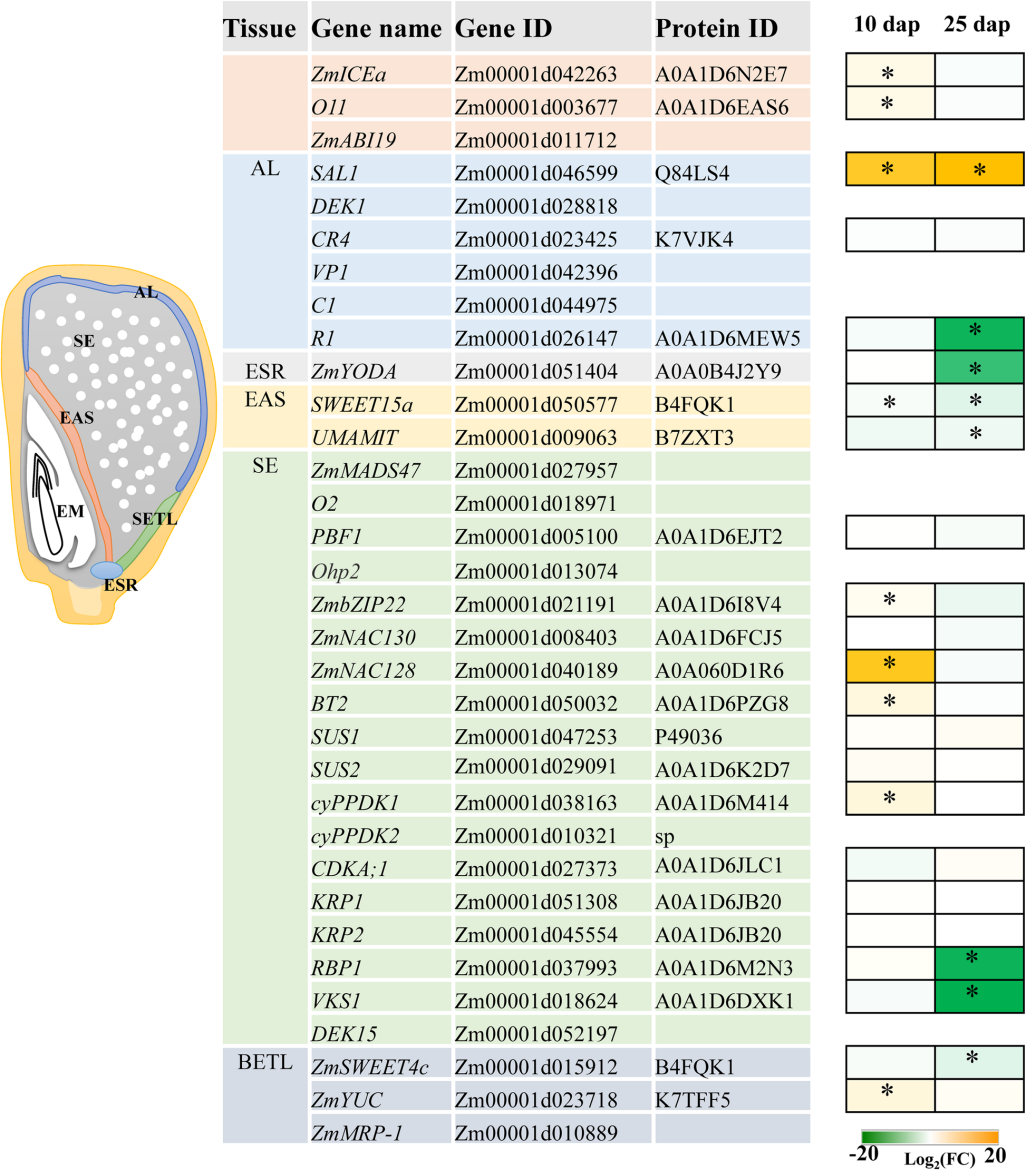
Effects of drought stress on endosperm development at proteomic level. A transcriptional regulatory network in the endosperm development shown (modified from Dai et al. 2021). The Log_2 _(fold change) was calculated for WW conditions relative to WS. SE: starchy endosperm, AL: aleurone, BETL: basal endosperm transfer layer, ESR: embryo-surrounding region, EAS: endosperm adjacent to scutellum. WW: well-watered; WS: water-stressed. Asterisks designate statistical (using Student’s t-test) significance *P* < 0.01

The main component of maize kernels is the endosperm, which includes starchy endosperm (SE), aleurone (AL), basal endosperm transfer layer (BETL), and embryo-surrounding region (ESR) cells. Moreover, the endosperm adjacent to scutellum (EAS) cells appears around 9 dap and persists throughout embryo growth [38]. Considering that DS affects the development of endosperm, we specifically explored the accumulation patterns of proteins related to endosperm development under WS conditions. Therefore, we collected 33 genes related to different cells of the endosperm from the report of Dai et al. [39] (Fig. 5). In the present study, A0A1D6EAS6 (*O11*) and A0A1D6N2E7 (*ZmICEa*), as central hub factors regulating endosperm development, were upregulated under WS treatment at 10 dap (Fig. 5). The proteins of Q84LS4, A0A1D6I8V4, A0A060D1R6, A0A1D6PZG8, A0A1D6M414, and K7TFF5, which involved in AL, EAS, SE, and BETL, respectively, were upregulated under WS treatment at 10 dap, but these proteins were reversed at 25 dap (Fig. 5). Proteins of A0A1D6MEW5, A0A0B4J2Y9, B4FQK1, B7ZXT3, A0A1D6M2N3, A0A1D6DXK1, and B4FQK1 were downregulated under WS treatment at 25 dap (Fig. 5).

In addition, we identified 21 proteins involved in starch biosynthesis and analyzed the accumulation patterns through proteomic data. Among these 21 proteins, 8 were differentially accumulated at 10 dap and 25 dap. The accumulation levels of A0A1D6NT44 (*sh1*), A0A1D6PXQ9 (*su1*), A0A1D6NHZ0 (*sh2*), Q947B9 (*agpsl1*), A0A1D6NUU8 (*ss1*), and A0A1D6EBS7 (*sbe4*), which associated with SUS, AGPase, SSS, and SBE, respectively, were upregulated under WS treatment at 10 dap. The proteins A0A1D6HWJ3 (*agpsl2*) and A0A1D6JEU3 (*ss2*), which associated with AGPase and SSS, were upregulated by DS at 25 dap. Only one protein, i.e., A0A1D6IAT2 (which related to UGPase), was downregulated under WS treatment at 25 dap (Figure S6). These results indicated that the changes in the proteome might accelerate endosperm development and kernel maturation and increase the accumulation of starch in the early stages of kernel development under DS.

## Discussion

### Dynamic response of kernel development to drought stress during grain filling

In the context of climate change, drought stress will have a profound impact on maize productivity in the future [40, 41]. During the whole growth period of maize, the grain-filling stage is important for kernel development and quality formation, and DS at this stage results in kernel abortion and yield reduction [42]. In the present study, DS after pollination inhibited kernel development (Fig. 1). From 25 to 30 dap, the kernel increase rate under WW treatment was significantly higher than that under WS treatment, indicating that kernel is close to maturity under WS. In addition, the changes in the kernel water content proved this point, and the kernel water content under WS treatment had a fast decline rate (Fig. 1C). Endosperm development affects kernel weight and quality. DS may have affected the development of endosperm cells and reduced the number of endosperm nuclei, resulting in obstructed grain filling and decreased yield [6, 7].

Thus, the molecular regulation mechanism of endosperm development is crucial for the formation of grain yield under DS. In this study, we collected 33 proteins related to endosperm development and analyzed their protein accumulation patterns under WW and WS treatments through proteomics. The upregulation of nine proteins that related to AL, EAS, SE, and BETL under WS treatment at 10 dap might jointly regulate the rapid development of kernels under WS treatment. For example, Feng et al. [43] reported that *ZmICEa* (which encodes A0A1D6N2E7) interacts with *O11* (which encodes A0A1D6EAS6), and they coregulate endosperm development and stress response. *ZmNAC128* (which encodes A0A060D1R6) positively regulates the accumulation of starch and protein in grains [44]. The TF *ZmbZIP22* (which encodes A0A1D6I8V4) regulates the 27 kD γ-zein gene expression and affects protein body initiation during endosperm development [45]. Moreover, the accumulation of seven other proteins were downregulated under DS at 25 dap, which may be consistent with the significant reduction in the fresh and dry weights of grains (*P* < 0.01). Feng et al. [43] reported that *O11* is a central regulator linking maize endosperm development, which regulates the expression of *ZmYODA* (which encodes A0A0B4J2Y9) specifically expressed in ESR. The maize *VKS1* (which encodes A0A1D6DXK1) regulates mitosis and cytokinesis during early-endosperm development, and its loss of function will lead to reduced cell proliferation and grain size [46]. These proteins may affect the rate of grain filling and shorten the grain-filling and ripening periods by affecting the endosperm development. Overall, these proteins may coregulate kernel development under WS conditions.

### Effects of drought stress on starch formation during the grain-filling stage

The most important nutrient stored in the endosperm of maize is starch, accounting for about 75% of the dry weight of grain. The morphological and physicochemical properties of starch granules affect the grain quality [11]. Numerous studies showed that the biosynthesis and accumulation of starch are remarkably influenced by DS [16, 47]. DS inhibits the activities of starch synthetic enzymes (such as AGPase, SSS, SBE, and GBSS), which suppress grain development and ultimately reduce grain weight [16, 48]. In present experiment, we found that the activities of AGPase, SUS, SBE, SDEB, and SSS were reduced by WS treatment during the grain-filling stage (Fig. 2). In addition, WS treatment decreased the starch accumulation from 20 to 30 dap, and the spherical shapes of starch granules were unchanged after the WS treatment (Fig. 1E). Li et al. [49] reported a similar phenomenon for starch granules under WS treatment during grain filling. In the endosperm development, the expression of starch biosynthesis proteins is also reported to be affected by DS [50, 51]. In this study, 21 proteins related to starch biosynthesis were found using proteomics (Figure S1). Compared with WW conditions, six DAPs (i.e., A0A1D6NT44, A0A1D6PXQ9, A0A1D6NHZ0, Q947B9, A0A1D6NUU8, and A0A1D6EBS7) were upregulated under WS treatment at 10 dap, and this finding might be related to the upregulation of proteins that regulated SE in kernel (Fig. 5). Notably, no difference in starch content was observed between WW and WS treatments at 10 dap, but the starch content under WS treatment was significantly higher than that under WW treatment at 15 dap (Fig. 1D). Our results showed that DS accelerated the accumulation of starch at early grain development stage. Therefore, these enzymes and proteins had coordinated participation in the complex starch biosynthesis in maize kernel under DS.

### Proteomic analysis of drought stress response during the grain filling

Understanding how kernels respond to drought stress at the molecular level is important for improving the maize yield and quality. In this study, proteomic changes at 10 dap and 25 dap were surveyed to gain insight into the protein accumulation patterns in kernel in response to DS (Fig. 3). We identified 487 and 465 DAPs at 10 dap and 25 dap, respectively. Among these DAPs, 21 and 25 TFs were identified at 10 dap and 25 dap, respectively (Fig. 3). In previous studies, TFs play important roles in DS response and plant development [52]. For example, the rice gene *OsICE1* (*OsbHLH002*), which is orthologous to A0A1D6N2E7 (upregulated at 10 dap), has been reported to improve tolerance to abiotic stresses in tobacco [53]. The accumulation of A0A1D6IHZ6 (an ortholog of *OsWRKY78*), a stem elongation and seed development regulator in rice [54], was downregulated at 25 dap. In our data set, four DAPs (catalase, glutamine synthetase, abscisic acid stress ripening 1, and lipoxygenase) were downregulated by DS (Figure S3). Catalase plays a key role not only in the defense against oxidative stress but also in governing cellular redox status and regulating cellular signaling [55]. The ectopic expression of the maize *Cat2* gene (A0A1D6JMR5) in tobacco plants changes the levels of catalase and increases the resistance to oxidative stress [56]. The glutamine synthetase (GS) activity and expression are reported to be modulated in various plants to respond to DS [28, 57]. James et al. [58] previously reported that *OsGS1;1* (a homolog of A0A1D6Q9W2), which overexpresses transgenic rice, enhances tolerance to osmotic stress and reduces ROS production. A previous study suggested that abscisic acid-, stress-, and ripening-induced (ASR) proteins are involved in the response of plant cell to various stressors [59]. The overexpression of *OsASR1* (a homolog of B4FKG5) in rice plants enhances salinity and drought tolerance and improves crop yields [60]. As a multifunctional enzyme, lipoxygenases play an important role when plants are subjected to biotic and abiotic stresses [61]. Consequently, catalase, glutamine synthetase, abscisic acid stress ripening 1, and lipoxygenase were downregulated under DS, which indicated that DS had a serious influence on kernel development.

The GO functional classification analysis revealed that upregulated and downregulated DAPs at the two stages were associated with a wide range of functions. The GO terms oxidation–reduction process (GO: 0,055,114) and oxidoreductase activity (GO: 0,016,672) were the most highly enriched among the downregulated DAPs at both stages (Fig. 4). These proteins were involved in the oxidation–reduction process, which also provide further information about the response of kernel development and starch formation responses to drought. In addition, 17 and 11 common DAPs were identified in upregulated and downregulated groups at 10 dap and 25 dap, respectively (Table 1). Among these data set, four proteins had homologous genes with known functions in rice (Table 1). Kim et al. [34] reported that *PDIL1-1* (a homolog of A0A1D6H543) controls endosperm development through the regulation of the amount and composition of seed proteins in rice. A recent study found that *OsADF2*-OE (a homolog of B4FTP0) maintains a relatively high relative water content, plant height, membrane stability index, and dry weight under DS [35]. *MODD* (a homolog of B6SLJ0) negatively regulates ABA signaling and drought tolerance in rice [36]. The phenotypic analysis of the *bs1* mutant revealed that *BS1* (a homolog of A0A1D6H5J5) plays an important role in the maintenance of proper acetylation level on the xylan backbone [37]. Consequently, these proteins may be important candidates for the endosperm development and DS response of the waxy maize kernel during grain filling.

## Conclusions

In summary, drought stress during grain filling seriously affected the physiological and biochemical parameters related to waxy maize kernel development and starch formation. Comparative proteome analysis identified 487 and 465 DAPs at 10 dap and 25 dap under drought stress, respectively. Among them, some proteins associated with endosperm development and starch biosynthesis were upregulated at 10 dap, which could be responsible for the shortened grain filling, and increased starch accumulation in the early stages of kernel development. Furthermore, some of the DAPs that were positively related to stress responses were downregulated in the waxy maize kernels and might negatively affect the drought tolerance of kernels. Notably, we determined four promising candidate proteins for kernel development and drought tolerance for subsequent functional validation. Our findings provided novel insights into the proteomic level and physiological response mechanism of waxy maize kernel development and starch formation under drought stress.

## Methods

### Plant materials and DS treatment

A pot trial was performed at Yangzhou University experimental farm (Yangzhou, China) in 2018–2019. The seeds of waxy maize ‘Suyunuo5’ (provided by Jiangsu Yanjiang Institute of Agricultural Sciences, Nantong, China), a widely planted waxy maize variety in Southern China, was selected as experimental material. Seeds with the same size were sown in seedling trays. Three 7-day-old healthy seedlings were transplanted into pots (diameter = 38 cm, height = 43 cm) containing 30 kg loam soil obtained from the field. At the jointing stage, a single plant was left in the pot, and the plants in all pots grew to the same state. Plants were provided a basal dressing of 10 g per pot (commercial fertilizer, N:P_2_O_5_:K_2_O = 15%:15%:15%) at the transplanting date and a top dressing of 6.6 g per pot (commercial urea, N = 46%) at the jointing stage.

All plants were grown under natural conditions until pollination. After pollination, these plants were moved to a mobilizable transparent waterproof shed and treated with water until maturity. The soil relative moisture contents under well-watered (WW) and water-stressed (WS) conditions were 75%–80% and 50%–55%, respectively. The amount of water loss was calculated and resupplied by weighing at 09:00 every day. These treatments were applied from 1 to 30 days after pollination (dap). After 30 dap, they were rewatered normally until maturity.

### Grain harvest and phenotypic identification

The ears of five plants were harvested at 5, 10, 15, 20, 25, and 30 dap. The kernels in the middle of the ear were used for analysis. The kernel dry weight was weighed after the kernel was dried in an oven at 80 °C for 24 h. The starch content was determined using the anthrone–sulfuric acid method with three biological replicates of each treatment [62]. The kernel water content (%) was calculated as: Kernel water content (%) = (fresh weight – dry weight)/fresh weight×100.

$$\mathrm{{Kernel}\,{water}\,{content}}(\mathrm{\%}) =\frac{\mathrm{{fresh}\, {weight}\,}-\mathrm{{dry}\,{weight}}}{\mathrm{{fresh}\,{weight}}}\times 100$$

### Scanning electron micrographs of endosperm cells

The resin slicing method was used to cut the kernels at 10, 15, 20, 25, and 30 dap into slices with thickness of about 300 mm (*n* = 5 per stage per treatment). After the slices were plated with ion-sputtered gold, endosperm cells and starch granules were observed using the GeminiSEM 300 (Carl Zeiss, Jena, Germany) environmental scanning electron microscope.

### Enzymatic activity assays for starch biosynthesis

Waxy maize kernels (middle position of ears) were stripped from ears at 5, 10, 15, 20, 25, and 30 dap and stored at a refrigerator maintained at − 80 °C. We assayed the activities of five starch biosynthesis enzymes, including SUS, AGPase, SSS, SBE, SPS and starch-debranching enzyme (SDBE), respectively, using the reagent kits ml10561, ml20341, ml10983, ml10724, ml056613 and ml10726 obtained from Shanghai Enzyme-linked Biotechnology Co., Ltd. All enzymatic activities were measured by enzyme-linked immunoassay, with three biological replicates. The OD value of SUS, AGPase, SSS, SBE, SPS and SDBE was measured with a microplate reader (Labsystems Multiskan, MS 352, Finland) at 450 nm and quantified using a standard curve.

### Protein extraction

Total proteins were extracted from the kernels under WW and WS treatments at 10 dap (10 days after treatment) and 25 dap (25 days after treatment) with three biological replicates in accordance, and the methods described by Zhu et al. [63] with minor modifications. Approximately 500 mg maize kernels were ground in liquid nitrogen, then dissolved with the lysis buffer and centrifuged at 25,000 g at 4 °C for 15 min. The supernatant was then transferred into a new tube and precipitated with 4 volumes of prechilled 10% TCA-acetone at –20 °C overnight and centrifuged at 25,000 g at 4 °C for 30 min. Pellets were washed by prechilled acetone three times. The protein dissolved with the lysis buffer and protein concentration was determined by the Bradford assay. Protein digestion was performed using the filter-aided sample preparation method with trypsin (Promega, Madison, WI) in 50 mM NH_4_HCO_3_ (Sigma, MO, USA) [64]. The absorbance was determined at A280 nm by using the NanoDrop 2000 instrument (Thermo Fisher Scientific, Mass, USA) to measure the peptide concentration. Resulting peptides were then separated and analyzed using the Orbitrap Fusion Tribrid mass spectrometer (Thermo Fisher Scientific, Mass, USA) equipped with the EASY-nLC 1200 system (Thermo Fisher Scientific, Mass, USA).

### Protein identification and bioinformatics analysis

The detailed protocol for data-dependent acquisition of library construction and data-independent acquisition (DIA) of peptide quantification were performed on the Orbitrap Fusion Tribrid mass spectrometer (Thermo Fisher Scientific, Mass, USA) equipped with the EASY-nLC 1200 system (Thermo Fisher Scientific, Mass, USA) in accordance with a previous method [65] with minor modifications. Samples (2 μg) were analyzed on the C18 trap column (150 μm I.D. × 25 cm, C18, 1.9 μm, 120 Å, Dr. Maisch GmbH). For the DIA analysis, the full scan was set at a resolution of 60,000 at *m/z* 350–1500. A total of 45 variable DIA windows were set for DIA acquisition ranging from *m/z* 200 to *m/z* 2000 with resolution of 30,000. Protein identification and quantification were finished using the Spectronaut pulsar X 12.0 (Biognosys) at default setting. In general, the raw spectral data were searched against the Uniprot (https://www.uniprot.org/, 2019.1.30) B73 complete proteome database (99,254 sequences). All identified proteins were filtered at 1% false discovery rates (FDRs). A DAP was defined with |log_2_ (fold change) |≥ 1 and FDR < 0.05. Functional annotations of DAPs were performed using the Gene Ontology (GO) annotation (http://www.geneontology.org/) and Kyoto Encyclopedia of Genes and Genomes (KEGG) pathway analyses of DAPs were based on KEGG database (https://www.kegg.jp/kegg/kegg1.html) [31]. The homologous genes of maize in rice (identity > 40% and coverage > 60%) were identified using the NCBI protein BLAST (https://blast.ncbi.nlm.nih.gov/Blast.cgi) [66].

We selected ten identified DAPs and analyzed the mRNA expression levels of corresponding genes by using quantitative real-time PCR (qRT-PCR). The qRT-PCR was performed on ABI ViiA™ 7 Real-Time PCR System (Applied Biosystems, CA, USA). Maize *GAPDH* was used as the internal reference gene [22]. The relative expression levels were calculated by the 2^−∆∆Ct^ method [22]. The Primer Premier 6 software was used to design primers on the basis of cDNA sequences in the maizeGDB (https://www.maizegdb.org/, Table S3).

### Statistical analysis

The statistical analyses of physiological data were performed with SPSS v19.0 using ANOVA, followed by LSD test to evaluate the significant differences at *P* < 0.01.

## Supplementary Information

**Additional file 1: Table S1**. Total proteins identified in waxy maize kernels.

**Additional file 2: Table S2**. Total differentially accumulated proteins identified at 10 dap and 25 dap.

**Additional file 3: Table S3**. Primer sequences of DAPs encoding genes used for qRT-PCR. **Figure S1. **Principal component analysis (PCA) of all proteins at 10 dap (A) and 25 dap (B) under WW and WS treatments. **Figure S2**. mRNA expression level analysis (qRT-PCR) of ten DAPs. Log_2_ (fold change) was calculated for WS conditions relative to WW. **Figure S3**. Classical maize genes among drought stress-responsive proteins. Log_2_ (fold change) of the proteins values was calculated for WS treatment relative to WW. **Figure S4**. KEGG pathway enrichment analysis of all DAPs at 10 dap and 25 dap [65]. **Figure S5**. GO functional classification of all DAPs at 10 dap and 25 dap. **Figure S6**. Relative proteins level from proteomic involved in starch biosynthesis. The Log_2_ (fold change) was calculated for WW conditions relative to WS. Asterisks designate statistical (using Student’s *t*-test) significance *P *< 0.01.

## Data Availability

The mass spectrometry proteomics data have been deposited to the ProteomeXchange Consortium (http://proteomecentral.proteomexchange.org) via the iProX partner repository [67] with the dataset identifier PXD026755.

## Abbreviations

AGPase: Adenosine diphosphate-glucose pyrophosphorylase
AL: Aleurone
BETL: Basal endosperm transfer layer
dap: Days after pollination
DAP: Differentially accumulated protein
DIA: Data-independent acquisition
DS: Drought stress
EAS: Endosperm adjacent to scutellum
ESR: Embryo-surrounding region
GO: Gene Ontology
GS: Glutamine synthetase
KEGG: Kyoto Encyclopedia of Genes and Genomes
PCA: Principal component analysis
qRT-PCR: Quantitative real-time PCR
SBE: Starch branching enzyme
SDBE: Starch-debranching enzyme
SE: Starchy endosperm
SPS: Sucrose phosphate synthase
SSS: Soluble starch synthase
SUS: Sucrose synthase
TF: Transcription factor
WS: Water-stressed
WW: Well-watered
