# Supplementary material for: Proteomics reveals the effects of drought stress on the kernel development and starch formation of waxy maize
Source: BMC Plant Biol. 2021 Sep 23;21:434. doi: 10.1186/s12870-021-03214-z (PMC8461923; doi:10.1186/s12870-021-03214-z)
Supplement: Supplementary file 3 — Additional file 3: Table S3. Primer sequences of DAPs encoding genes used for qRT-PCR. Figure S1. Principal component analysis (PCA) of all proteins at 10 dap (A) and 25 dap (B) under WW and WS treatments. Figure S2. mRNA expression level analysis (qRT-PCR) of ten DAPs. Log2 (fold change) was calculated for WS conditions relative to WW. Figure S3. Classical maize genes among drought stress-responsive proteins. Log2 (fold change) of the proteins values was calculated for WS treatment relative to WW. Figure S4. KEGG pathway enrichment analysis of all DAPs at 10 dap and 25 dap [65]. Figure S5. GO functional classification of all DAPs at 10 dap and 25 dap. Figure S6. Relative proteins level from proteomic involved in starch biosynthesis. The Log2 (fold change) was calculated for WW conditions relative to WS. Asterisks designate statistical (using Student’s t-test) significance P < 0.01. [file 12870_2021_3214_MOESM3_ESM.pdf]

Table S3. Primer sequences of DAPs encoding genes used for qRT-PCR.

Primer name	Sequence (5'-3')
Zm00001d014814F	TTCAAGAACGCTGGCTATGG
Zm00001d014814R	TCGAAGTGGGTTATGATGGG
Zm00001d012572F	GACCTCCAAAGCCTGATAAGTG
Zm00001d012572R	GAACCTTGTAGCTTCCGCACT
Zm00001d017640F	TCTCAGGAATATGGTGGTGGG
Zm00001d017640R	CAGAATGCTGCGTAAGTGTCG
Zm00001d021596F	TTTCCCACCGTGCTCCTGTT
Zm00001d021596R	GTCCCCTCCACCTCCCTTCT
Zm00001d016130F	ACGCTGGCAACCTCATCC
Zm00001d016130R	GCGTTCTTGGCCTTGGAC
Zm00001d006157F	AAAGCAGCATCGTCGCAGAG
Zm00001d006157R	CGACAATCTTGCGGCTCATA
Zm00001d042263F	CCAAGCCCTACTGGACAACAA
Zm00001d042263R	CACAGACCTCAGCTCGGAACA
Zm00001d003677F	GCTCCATGACTGCGACGAC
Zm00001d003677R	AGCTTGGTCCTGGCTTTGTT
Zm00001d045893F	GATGGCGATGTTAGTCTTGATGG
Zm00001d045893R	GATTGCTGTCCTGTAGAATTTGC
Zm00001d021191F	AGAGTGCCACCTCTGTCTCCA
Zm00001d021191R	ACTTCACCTTGCGCTCCTTA
GAPDH-F	CCCTTCATCACCACGGACTAC
GAPDH-R	AACCTTCTTGGCACCACCCT

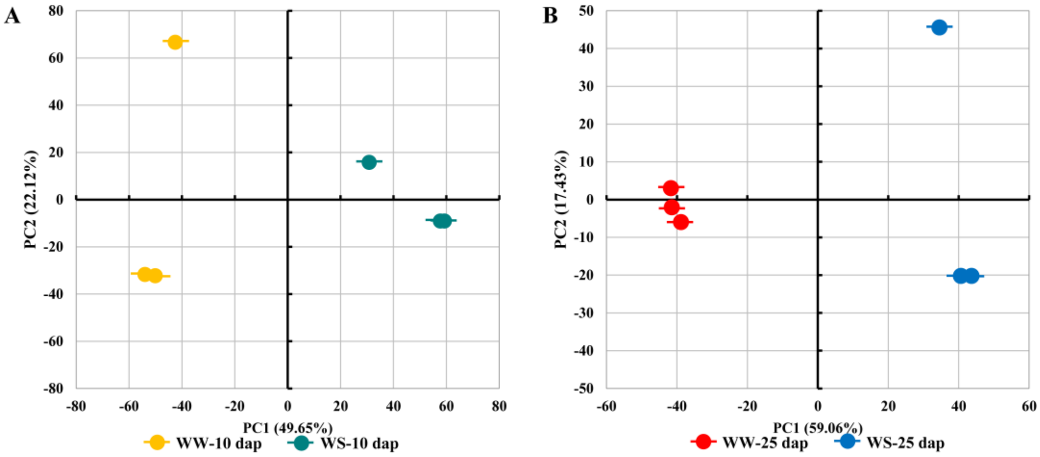

Figure S1. Principal component analysis (PCA) of all proteins at 10 dap (A) and 25 dap (B) under WW and WS treatments.

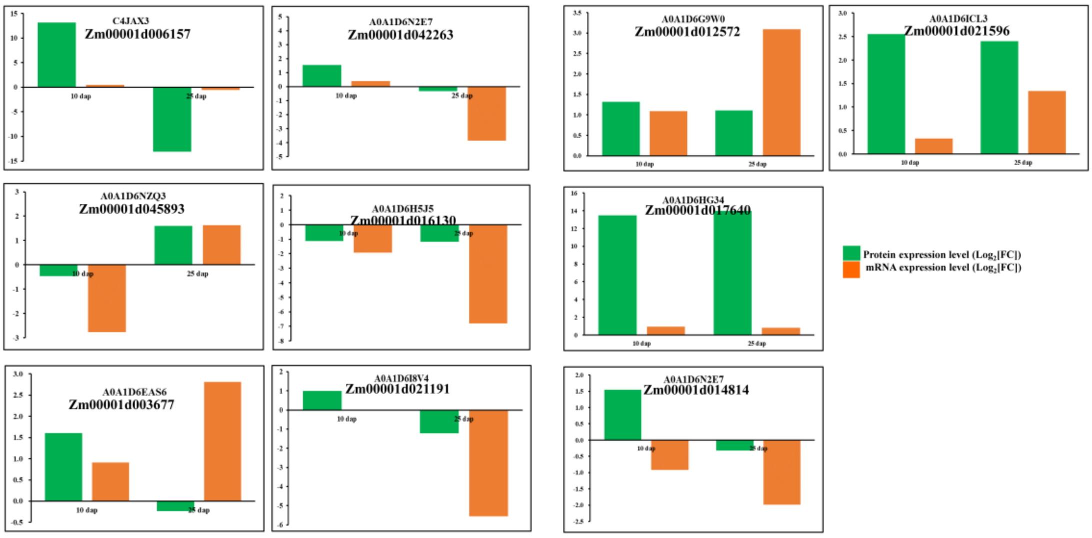

Figure S2. mRNA expression level analysis (qRT-PCR) of ten DAPs. Log_2 (fold change) was calculated for WS conditions relative to WW.

10 dap

Protein ID	Gene ID	Gene name	Description
A0A1D6HWR9	Zm00001d019312	*bgaf1*	Beta glucosidase aggregating factor 1
A0A1D6JMR5	Zm00001d027511	*cat2*	Catalase
B6TJA9	Zm00001d034635	*chi1*	Chalcone-flavonone isomerase family protein
C0P9T6	Zm00001d044673	*cyp1*	Sterol 14-demethylase
A0A1D6MJV0	Zm00001d039631	*gl13*	Glossy13
C0PGM3	Zm00001d033447	*glb1*	Globulin-1 S allele
A0A1D6Q9W2	Zm00001d051804	*gln5*	Glutamine synthetase
A0A1D6GPW8	Zm00001d014100	*grp3*	Glycine rich protein 3
A0A1D6L4S2	Zm00001d034035	*gsht1*	Glutathione transporter 1
A0A1D6EH08	Zm00001d004687	*hsbp1*	O-methyltransferase ZRP4
A0A1D6IP14	Zm00001d022557	*nas5*	Nicotianamine synthase 5
A0A1D6E5Y9	Zm00001d003016	*pal2*	Phenylalanine ammonia-lyase
Q6RXY1	Zm00001d028733	*pza03243*	Non-specific serine/threonine protein kinase
B4FBA4	Zm00001d048373	*wc1*	9,10-9,10 carotenoid cleavage dioxygenase 1
B6T9S4	Zm00001d007403	*whp1*	Chalcone synthase
A0A1D6IYZ9	Zm00001d024386	*xth1*	Xyloglucan endo-transglycosylase/hydrolase 1
A0A1D6H9T3	Zm00001d016684	*ae1*	1,4-alpha-glucan branching enzyme
Q947B9	Zm00001d032385	*agpsl1*	Glucose -1- phosphate adenylyltransferas
K7TWQ8	Zm00001d050567	*als1*	Acetolactate synthase
A0A1D6GRB4	Zm00001d014258	*alt4*	Alanine aminotransferase 5
A0A1D6N2E7	Zm00001d042263	*bhlh175*	Inducer of CBF expression 2
B4F832	Zm00001d015746	*bt1*	Adenine nucleotide transporter BT1 chloroplastic/mitochondrial
A0A1D6PZG8	Zm00001d050032	*bt2*	Glucose-1-phosphate adenylyltransferase
A0A1D6I8V4	Zm00001d021191	*bzip22*	Basic-leucine zipper (BZIP) transcription factor family protein
A0A1D6MK18	Zm00001d039658	*bzip6*	Transcription factor HY5-like
K7V794	Zm00001d045431	*eno1*	Phosphopyruvate hydratase
A0A1R3N7W5	Zm00001d003398	*fl1*	Floury endosperm 1
B4FV70	Zm00001d013725	*ga20ox4*	Gibberellin 20-oxidase4
Q946V3	Zm00001d038597	*glb3*	Alpha globulin
B4FUH2	Zm00001d043382	*got1*	Aspartate aminotransferase
A0A1D6H679	Zm00001d016198	*got3*	Aspartate aminotransferase
A0A1D6HCF4	Zm00001d017121	*gpc4*	Glyceraldehyde-3-phosphate dehydrogenase4
A0A1D6FKW5	Zm00001d009646	*gras20*	Scarecrow-like1
B6STA5	Zm00001d031168	*grp1*	Glycine-rich RNA-binding protein 2
A0A1D6JC04	Zm00001d026056	*hyd3*	Hydroxylase 3
A0A1D6MYR1	Zm00001d041847	*mybr14*	Switch/sucrose nonfermenting 3C
K7TTR0	Zm00001d049610	*orp1*	Tryptophan synthase
K7TR93	Zm00001d024702	*orp2*	Tryptophan synthase
A0A1D6M414	Zm00001d038163	*pdk1*	Pyruvate, phosphate dikinase
A0A1D6FQH6	Zm00001d010321	*pdk2*	Pyruvate orthophosphate dikinase 2
A0A1D6KID1	Zm00001d031332	*pza03531*	Heat shock protein 90-2
A0A1D6GKL8	Zm00001d013568	*rab15*	Glycine-rich RNA-binding, abscisic acid-inducible protein
A0A1D6EBT5	Zm00001d003822	*s1fa1*	DNA-binding protein S1FA
A0A1D6IWN4	Zm00001d023892	*sad1*	Shikimate dehydrogenase
A0A1D6EBS7	Zm00001d003817	*sbe3*	Starch branching enzyme IIa
A0A1D6NT44	Zm00001d045042	*sh1*	Sucrose synthase
A0A1D6NHZ0	Zm00001d044129	*sh2*	Glucose-1-phosphate adenylyltransferase
A0A1D6KL30	Zm00001d031727	*sodh1*	Sorbitol dehydrogenase
A0A1D6NUU8	Zm00001d045261	*ss1*	Starch synthase, chloroplastic/amyloplastic
A0A1D6PXQ9	Zm00001d049753	*su1*	Sugary1
A0A1D6LVT4	Zm00001d037234	*su2*	Starch synthase, chloroplastic/amyloplastic
A0A1D6Q673	Zm00001d051316	*thx16*	Putative homeodomain-like transcription factor superfamily protein isoform 1
A0A1D6QCI5	Zm00001d052060	*trps10*	Trehalose-phosphatase
B6TI69	Zm00001d018969	*tsa1*	Tryptophan synthase

25 dap

Protein ID	Gene ID	Gene name	Description
B4FKG5	Zm00001d023529	*aasr1*	Abscisic acid stress ripening1
B6TK34	Zm00001d038208	*alf5*	PHD finger protein
A0A1D6MYY5	Zm00001d041882	*atp1*	V-type proton ATPase proteolipid subunit
B6UC65	Zm00001d048703	*bx4*	Benzoxazinone synthesis4
A0A1D6I8V4	Zm00001d021191	*bzip22*	Basic-leucine zipper (BZIP) transcription factor family protein
A0A1D6GWA0	Zm00001d014773	*cdpk1*	Calcium dependent protein kinase1
K7V3X5	Zm00001d053016	*cdpk12*	Putative calcium-dependent protein kinase family protein
A0A1D6MK23	Zm00001d039660	*cesa3*	Cellulose synthase
A0A1D6EPE8	Zm00001d005675	*emp2*	Pentatricopeptide repeat-containing protein mitochondrial
A0A1D6KCB1	Zm00001d030373	*eno2*	Phosphopyruvate hydratase
A0A1D6KJS7	Zm00001d031593	*fha2*	FHA domain-containing protein FHA2
A0A1D6HY98	Zm00001d019536	*glk15*	Protein PHOSPHATE STARVATION RESPONSE 1
A0A1D6E965	Zm00001d003429	*gpa1*	Glyceraldehyde-3-phosphate dehydrogenase
A0A1D6DVH5	Zm00001d002000	*lox6*	Lipoxygenase
B6TIF9	Zm00001d042843	*na1*	Steroid 5-alpha-reductase DET2
A0A1D6M0N9	Zm00001d037811	*PCO134814*	Proline-rich receptor-like protein kinase PERK1
A0A1D6JS18	Zm00001d028098	*phm3226*	Radical SAM superfamily protein
B8A2W4	Zm00001d033800	*ptr1*	Protein NRT1/ PTR FAMILY 8.3
A0A1D6K7D0	Zm00001d029714	*pza00752*	SWIRM domain-containing protein
A0A1D6E202	Zm00001d002580	*pza02337*	Mechanosensitive ion channel protein 1 mitochondrial
B4FWF2	Zm00001d048086	*rfa1*	Replication protein A subunit
A0A1D6QTA8	Zm00001d053899	*rop2*	Rho-related protein from plants2
A0A1D6MA23	Zm00001d038728	*trps12*	Alpha,alpha-trehalose-phosphate synthase (UDP-forming)
A0A1D6M2S4	Zm00001d038023	*wrky83*	Putative WRKY DNA-binding domain superfamily protein
K7V5D2	Zm00001d011813	*bss1*	Bundle sheath strands specific1
A0A1D6JMR5	Zm00001d027511	*cat2*	Catalase
B4FDN2	Zm00001d046450	*dbptf4*	Protein-serine/threonine phosphatase
A0A1D6QG81	Zm00001d052316	*fer1*	Ferritin
A0A1D6DZW5	Zm00001d002353	*gl2*	Orf protein
A0A1D6E931	Zm00001d003401	*grf1*	14-3-3-like protein GF14-6
A0A1D6MY32	Zm00001d041772	*gst18*	Glutathione transferase
A0A1D6G2Y3	Zm00001d011680	*kelp3*	RNA polymerase II transcriptional coactivator KELP
K7VAM9	Zm00001d045310	*mpk2*	Mitogen-activated protein kinase
A0A1D6L548	Zm00001d034059	*mta1*	60S ribosomal protein L13a-1
B4FNC6	Zm00001d039250	*nlp1*	RWP-RK transcription factor
A0A1D6QPU6	Zm00001d053453	*pep3*	Phosphoenolpyruvate carboxylase isoform 1
A0A1D6GK60	Zm00001d013505	*prc2*	Proteasome subunit beta
A0A1D6LHK3	Zm00001d035616	*pza02948*	Inorganic diphosphatase
K7U5A5	Zm00001d053090	*pza03081*	14-3-3-like protein
A0A1D6HBA7	Zm00001d016928	*serk2*	Somatic embryogenesis receptor kinase 2
B4FH44	Zm00001d019034	*trh2*	Thioredoxin
B6T9S4	Zm00001d007403	*whp1*	Chalcone synthase
A0A1D6IHZ6	Zm00001d021947	*wrky1*	Putative WRKY transcription factor 20
A0A1D6KXG4	Zm00001d033291	*wrky102*	Prefoldin subunit 4
A0A1D6LNQ3	Zm00001d036494	*zim20*	GATA transcription factor 24

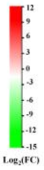

Figure S3. Classical maize genes among drought stress-responsive proteins. Log_2 (fold change) of the proteins values was calculated for WS treatment relative to WW.

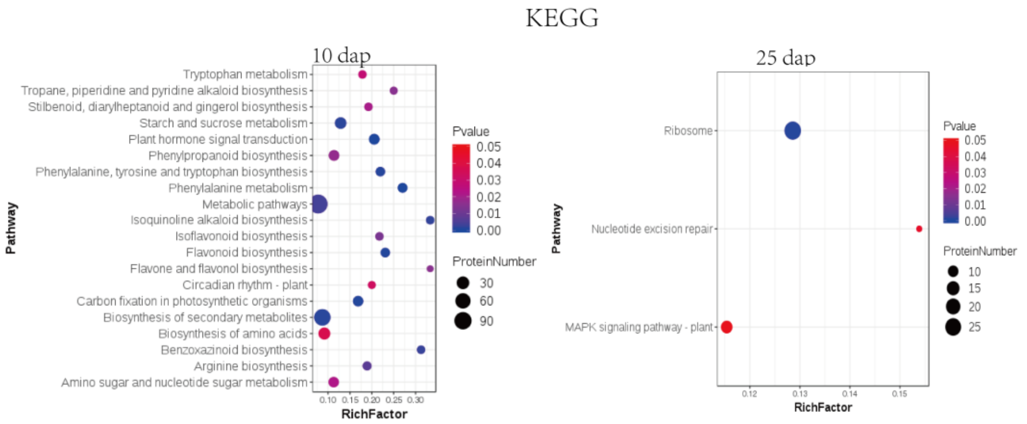

Figure S4. KEGG pathway enrichment analysis of all DAPs at 10 dap and 25 dap [65].

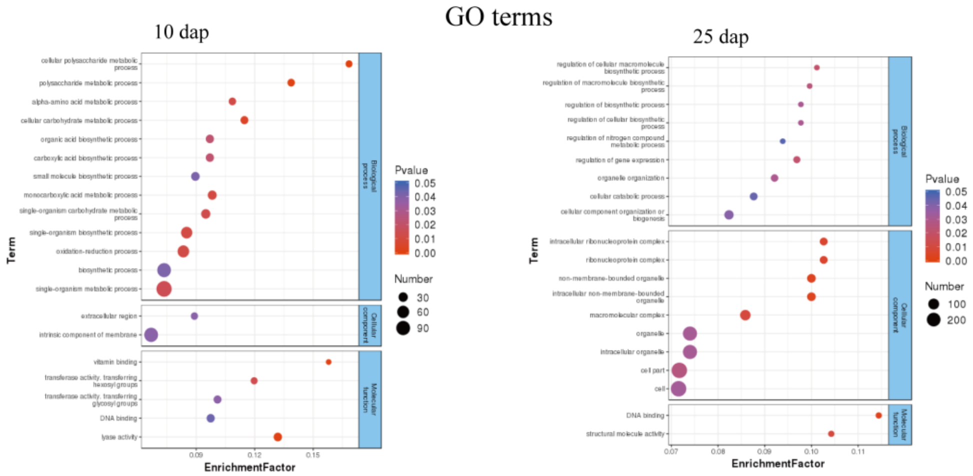

Figure S5. GO functional classification of all DAPs at 10 dap and 25 dap.

	Gene name	Gene ID	Protein ID
SUS	*spp1*	Zm00001d010523	A0A1D6FRK7
	sps1	Zm00001d012036	P31927
	sxd1	Zm00001d015985	C0HHY8
	sut2	Zm00001d041192	A0A1D6MV11
	sps2	Zm00001d042353	A0A1D6N358
	sh1	Zm00001d045042	A0A1D6NT44
	sus1	Zm00001d047253	P49036
	sut7	Zm00001d048311	K7WGT4
	su1	Zm00001d049753	A0A1D6PXQ9
	sh2	Zm00001d044129	A0A1D6NHZ0
UGPase	*ugp1*	Zm00001d006309	A0A1D6EUQ2
		Zm00001d021421	A0A1D6IAT2
AGPase	*agpll2*	Zm00001d019266	A0A1D6HWJ3
	agpsl1	Zm00001d032385	Q947B9
	agpll1	Zm00001d033910	A5GZ73
SS	*ss1*	Zm00001d045261	A0A1D6NUU8
	ss2	Zm00001d002256	A0A1D6JEU3
	ss4	Zm00001d010821	K7V1M1
SBE	*sbe4*	Zm00001d003817	A0A1D6EBS7
	sbei1	Zm00001d011685	A0A1D6G2Z4
	sbe1	Zm00001d014844	A0A1D6GWP4
	sbei2	Zm00001d043703	A0A1D6NEF2
SDBE	*zpu1*	Zm00001d004438	A0A1D6EFE0
	iso3	Zm00001d020799	A0A1D6I697
	su4	Zm00001d038121	A0A1D6M3H8

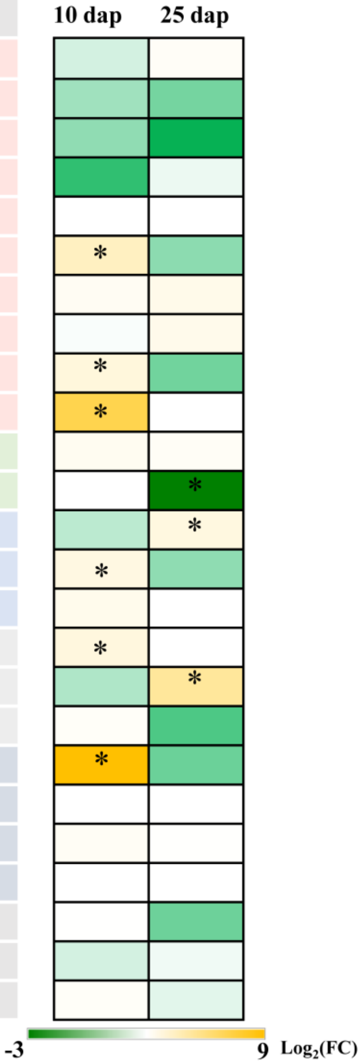

Figure S6. Relative proteins level from proteomic involved in starch biosynthesis. The Log_2 (fold change) was calculated for WW conditions relative to WS. Asterisks designate statistical (using Student's *t*-test) significance $P < 0.01$.
